# Detecting deception using machine learning with facial expressions and pulse rate

**DOI:** 10.1007/s10015-023-00869-9

**Published:** 2023-04-28

**Authors:** Kento Tsuchiya, Ryo Hatano, Hiroyuki Nishiyama

**Affiliations:** Department of Industrial Administration, Graduate School of Science and Technology, Tokyo University of Science, 2641 Yamazaki Noda, Chiba Japan

**Keywords:** Machine learning, Random forest, Deception detection, Facial information, Pulse rate

## Abstract

Given the ongoing COVID-19 pandemic, remote interviews have become an increasingly popular approach in many fields. For example, a survey by the HR Research Institute (PCR Institute in Survey on hiring activities for graduates of 2021 and 2022. https://www.hrpro.co.jp/research_detail.php?r_no=273. Accessed 03 Oct 2021) shows that more than 80% of job interviews are conducted remotely, particularly in large companies. However, for some reason, an interviewee might attempt to deceive an interviewer or feel difficult to tell the truth. Although the ability of interviewers to detect deception among interviewees is significant for their company or organization, it still strongly depends on their individual experience and cannot be automated. To address this issue, in this study, we propose a machine learning approach to aid in detecting whether a person is attempting to deceive the interlocutor by associating the features of their facial expressions with those of their pulse rate. We also constructed a more realistic dataset for the task of deception detection by asking subjects not to respond artificially, but rather to improvise natural responses using a web camera and wearable device (smartwatch). The results of an experimental evaluation of the proposed approach with 10-fold cross-validation using random forests classifier show that the accuracy and the F1 value were in the range between 0.75 and 0.8 for each subject, and the highest values were 0.87 and 0.88, respectively. Through the analysis of the importance of the features the trained models, we revealed the crucial features of each subject during deception, which differed among the subjects.

## Introduction

With the recent trends of digital transformation and the ongoing COVID-19 pandemic, remote (or online) interviews and relevant applications have become increasingly popular. These provide more opportunities to talk among participants who may be geographically dispersed. Typical application fields of them include commercial/enterprise communications, medical services, education, law enforcement and national security. For example, according to a survey of 240 companies conducted by the HR Research Institute [4], more than half of Japanese companies and 80% of large companies are utilizing remote interviews.

One of the challenging and significant tasks in (remote) interviews is deception detection. For some reason, an interviewee might attempt to deceive an interviewer or feel difficult to tell the truth. Deception detection might help interviewees who would like to tell the truth or be asked the right question. Such people might exist as a victim (or prosecution witness) of a crime in a court, a shy student who is asked a hard question to answer by his/her teacher, and a patient suffering from psychological disorders. In addition, deception detection might also assist interviewers who often need to conduct the risk assessment of (unfamiliar) interviewees [5]. Such people can be found in the human resources department of a company, border control in an airport, and a psychiatry department of a hospital.

In some cases, detected deception has a negative impact on the relevant process of interviews. For example, a survey published in the Journal of Job Hunting in 2018 [8] showed that approximately 70% of interviewers detected deception among applicants, and more than one-third of detected cases affected a negative impact. Therefore, the ability of interviewers to detect deception of interviewees is significant for their company or organization.

However, Bond et al. [2] pointed out that the average capability of untrained interviewers does not significantly exceed the chance of a lucky guess. They conducted a meta-analysis of over 200 studies on deception detection, and revealed that the probability of ordinary people successfully detecting deception was approximately 52%. There are several arguments regarding the difficulty of human deception detection, one of which is that humans have inherent biases [5]. For example, we tend to judge other’s statements as true regardless of their actual deliberations, and we tend to see others as liars if we view them as such. These biases also affect trained individuals, depending on their experience and the actual situation. Another possible reason is that we cannot fully grasp the phenomena that are characteristic of deception.

From the above background, the following two possible issues that should be overcome can be noted. First, interviewers may well detect deception incorrectly, accusing innocent interviewees. Second, some interviewers may not be able to detect deception. Humans naturally vary considerably in their ability to detect deception or lying, and in some cases, the interviewees may be at a disadvantage.

These indicate the need for accurate and unbiased automated deception detection systems. Note that, we should only use such systems as part of human in the loop system, to avoid ethical issues and unintended violations of local laws [5]. In other words, it is strongly required to classify the right suspects as deceptive without misclassifying innocent people.

Previous studies on deception detection that use image recognition have shown that the features of human nonverbal behaviors, such as changing facial expressions and pulse rate, are important factors in detecting deception, which seems to coincide with common sense. For example, in 2016, Watanabe et al. [9] claimed that changes in pulse rate are an important feature indicating human deception. In 2018, Wu et al. [10] showed that utilizing features based on facial expressions and voices of deceptive speakers was important, and later in 2021, Khan et al. [5] revealed that deceptive subjects showed more intense facial micro-movements associated with eyes during their deception.

However, to the best of our knowledge, no machine learning approach has been developed to associate features of facial expressions with pulse rate. One possible reason is that acquiring pulse rate requires wearing a wearable device, such as a smartwatch, which might cause discomfort. In addition, from a technical perspective, wearable devices designed to acquire pulse rate information accurately tend to be relatively small, because they need to operate at low frequencies to save battery power. For example, the Polar M600, which is an ordinary consumer smartwatch used in this study, can output only an estimated and averaged pulse rate per second.

Regarding more advanced approach, studies on estimating the pulse rate from facial expressions using, for example, a web camera have been conducted recently [11]. However, these methods are still under development; the methods used in the mentioned study were not able to estimate pulse rate remotely in real time.

Hence, we restrict our attention to establishing a bare-bones machine learning approach that associates the features of facial expressions and pulse rates using videos and wearable devices to detect deceptions automatically.

In this study, we propose a method to detect whether an interviewee is attempting to deceive an interlocutor by combining the interviewees’ nonverbal behaviors and biometric data, such as facial landmarks and pulse rate, using machine learning that allows us to exclude human bias from detection. We also present a method to construct our desired dataset from subjects by providing an environment that allows them to improvise deception naturally. The key idea of this method is based on the characteristics of remote interviewing, that is, the facial expressions of interviewees can be handled as data through their screen, and may involve a similar psychological situation without using role-played interviewing.

Our results may help support the decision-making process of (remote) interviewers by detecting deception from the facial expressions of interviewees, where they need to be careful with ethical issues and local laws for such an application.

## Related work

Wu et al. [10] developed a “deception analysis and reasoning engine,” which is a system for deception detection based on multi-modal information about humans available in a video. They used video data from a courtroom trial and trained various classifiers such as kernel support vector machine (SVM), naive Bayes classifier, and random forests. Eventually, the best performance among the models was an area under the curve (AUC) of approximately 87%, based on facial micro-movements, voice and some textual information. They also revealed that subtle lip and eyebrow movements are important features for classifying truthful and deceptive behaviors.

Mathur et al. [6] attempted to find significant differences in valences and arousal which are dimensional representations of facial emotions between truthful and deceptive speakers. Similar to [10], they also used actual video recordings of courtroom trial for their experiments. The deep learning library OpenFace was used to extract facial features from the video. As a result of the experiment, they achieved an AUC of 91% using emotional, visual, audio, and verbal features. In addition, they claimed that their result contributes computational support to the leakage hypothesis and the four factor theory in psychology.

Khan et al. [5] focused on a machine learning approach for deceptive detection using features of non-verbal behavior (NVB) and identified the features that are particularly important among them such as facial micro-movements, changes in gaze, and blink rates. Since their objective was to support EU border guards, the data used in their experiments were collected from subjects who were asked to simulate terrorists who attempted to deceive the guards. They achieved an accuracy of approximately 80% using random forests and revealed that the features of eye movements of role-played subjects were significant in deception detection.

However, there might be a restriction on their result owing to the unnatural role-playing approach of the subjects. In this study, we present an approach to data collection that allows subjects to naturally improvise deceptive behaviors.

## Proposed method

Our method follows an ordinary machine learning approach for image recognition. We describe the details of our method in this section, including data collection, labelling, feature extraction, preprocessing to construct the data set, and classification using machine learning.

### Data collection

The proposed approach is designed to detect deceptive statements made by interviewees in remote interviews using machine learning. To achieve this goal, we constructed a data acquisition environment. In a naive approach, we may consider to ask subjects to play the role of interviewees, e.g., students who were tested on whether he/she understood the contents of a certain lecture or job applicants, and respond to questions from an experimenter playing the role of an interviewer.

However, we considered that experimenters may not have sufficient experience to play this role realistically. Thus, maintaining a sense of realism and suspension of disbelief to generate a realistic mood would be difficult. Even worse, it might lead to unrealistic results. Although if we asked the subjects to exhibit deceptive behavior, the quality of the data may deteriorate. For example, the series of questions asked in the interviews are not disclosed by many organizations and companies; hence, the experimenters themselves need to create suitable questions. This approach makes it difficult to collect sufficient data for machine learning. For these reasons, setting up an environment to simulate actual interviews realistically is challenging. This would apply to any such role-playing encounter.

In this context, we consider that interviewees may make deceptive statements in (remote) interviews, such as falsifying their claims or backgrounds, to gain an advantage in the interviewing process by deceiving the interviewer. Interviewees may make deceptive statements to deceive the interviewer in response to questions.

Based on this observation, we adopted an alternative approach to obtain a sufficient amount of data by conducting an experiment that naturally provides a similar situation to the above, even if we do not simulate an actual interview. Therefore, we designed the following method to allow subjects to deceive the experimenter naturally.

First, we randomly displayed an image of the subject. The images were selected from a wide range of genres that most people would be expected to have some familiarity with, such as historical buildings, anime characters, and photographs of famous people. Then, we let the subject talk freely for a few minutes about the displayed image, where they were instructed to make deceptive statements freely. Meanwhile, we recorded their pulse rate and facial images using a smartwatch and a web camera, respectively. We continued this process until a sufficient amount of data was obtained. The ground-truth with respect to the deception of each utterance during the experiments was provided by the subjects after each experiment. The details, including assignment, are described in the next section.

### Labeling

**Fig. 1:**
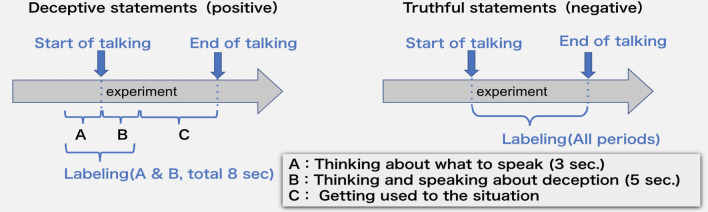
Definition of labels and corresponding periods of data

In this study, utterances were labeled depending on the subject’s intention. After each experiment, we asked the subject whether their utterance was deceptive one-by-one using a recorded video of the experiment.

Although various studies have defined many types of deception, the present study followed the definition of Hosomi et al. [3]. They defined deception as “the act of trying to make another person have a belief or understanding that the deceiver considers false.” The significant point here is that a deception is an intentional act; hence, false memory, ignorance, and errors are not regarded as deception.

We defined the subjects’ utterances as deceptive if they tried to convince the experimenter of something that the subject thought was false; otherwise, their statements were considered truthful. Then, we assigned the label of the positive case to the recorded data corresponding to the period during which the subject made a deceptive utterance, from 3 s before the subject began to speak (based on the time they opened their mouth) to 5 s immediately after that (see also the range of parts A and B on the left side of Fig. 1). The reason for using this definition of label assignment is as follows.

(i) Often, making appropriate decisions is difficult for subjects if they did not know what to say in advance. We expected that some signs (or features) would appear in their facial expressions or biometric information during this period.
(ii) We also thought that if the subject was thinking about the unknown content during the first few seconds of the deceptive utterance but subsequently became accustomed to the situation, the feature might disappear.
(iii) We found that that the range of 3 s before and 5 s after they began to speak deceptively yielded better results in several trials of our preliminary experiment and its analysis.

We also assigned the label of a negative case to the data corresponding to the range other than the above positive cases, that is, the entire period of speaking truthfully and the C part in Fig. 1, where the subject was speaking deceptively.

### Feature extraction

#### Landmark acquisition using OpenFace

First, we provide an overview of the techniques used to acquire the base information of a subject’s facial expression from the videos recorded in our experiment. Note that the biometric information, such as pulse rate, can be obtained directly from the smartwatch used in our experiment, so we omit these details.

**Fig. 2:**
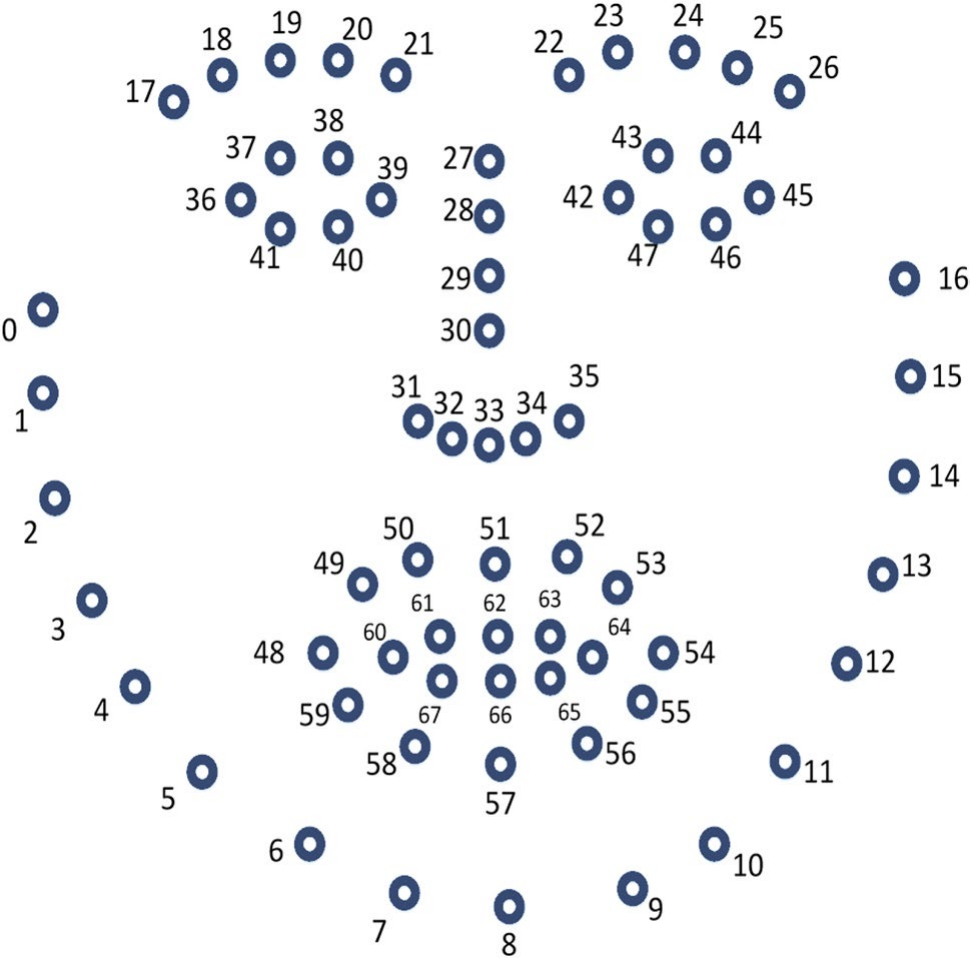
Example of landmarks by OpenFace

**Fig. 3:**
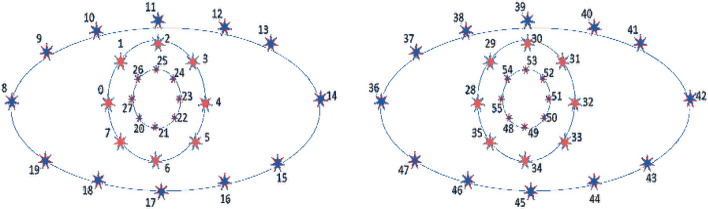
Example of landmarks with respect to subject’s eyes

For each frame of the recorded videos, we acquired information to extract facial features such as head posture, gaze, and a set of coordinates called landmarks from the image of a subject’s face. To acquire such information, we used a deep learning library called “OpenFace,” developed by the Multicomp group at Cambridge University [1]. OpenFace can be used to conduct facial behavior analysis such as landmark detection, head posture estimation, facial expression recognition, and gaze estimation in real time. We show example of landmarks retrieved using OpenFace in Fig. 2 and eye-specific landmarks in Fig. 3.

#### Overview of extracted features

Table 1 lists the features extracted in this study. We extracted some facial features from the acquired facial landmarks following the same method as described in [7]. In addition, we defined and calculated the following features that form the facial expressions of a subject, including eyebrow movement and tilt, eye movement and its area, mouth area, size of the corners of the mouth, and blink rate. Eye movement is expressed by the amount of time variation in the coordinates of the center of the eye (iris). To consider changes in the time series over the entire dataset, we also used the variance for 3 s before and after each time step, that is, for a total of 6 s.

For the features of biometric information, we used the pulse rate obtained from a smartwatch. In particular, we used the variance of the pulse rate for 10 s before and after for a total of 20 s. The range used to compute the variation in pulse rates differs from that of facial features, because pulse rate does not change as rapidly, according to the experience of our preliminary experiment.

**Table 1:** Ex﻿tracted fea﻿tures

Features extracted from points of Eyebow and Eyes	
Eyebrow tilt (right and left)	Distance between eyebrows and eyes (right and left)
Area between eyebrows	Area of the eyes (right and left)
Eye aspect ratio (left to right)	Blink rate
Eye movement (right, left, horizontal and vertical)	

Features extracted from points of Mouse	
Mouth area (inside/outside)	Mouth aspect ratio (inside/outside)
Degree of raise for mouth corners	Angle of mouth
Number of times the mouth is closed	

Features obtained from devices via provided library	
Gaze	Head tilt
Pulse rate	

In the following section, we describe the outline of the calculation for each feature shown in Table 1, where we denote the coordinates from 0 to 67 in Fig. 2 and 3 by points *P*0 to *P*67. To avoid confusion, we explicitly designate the figure name at the beginning of the exposition of features if we refer to the points in Fig. 3; otherwise, they are implicitly designated in Fig. 2. 

**Eyebrow tilt (left and right)** The slope of the right eyebrow (left eyebrow) was calculated from points $\{P17, P18, P19, P20, P21\}$ ($\{P22, P23, P24, P25, P26\}$) using the least squares method.

**Distance between eyebrows and eyes(right and left) ** The distance between the eyebrows and the eyes is the average length of the eight line segments between them. For example, we created the set $\{(P18, P36),$ $(P19, P37), (P20, P38), (P21, P39)\}$ of pairs of points on the right eyebrow and upper eyelid and then calculated the average length of these pairs as the distance between the right eyebrow and right eye. Similarly, we also calculated the distance between the left eyebrow and the left eye using the set $\{(P22, P42),$ $(P23, P43), (P24, P44), (P25, P45)\}$ of points. To ensure that the features were unaffected by the actual size of the face image, they were re-scaled using normalization, that is, the distance is divided by the length *L* of the nasal bridge, which is the distance the four points $\{P21, P22, P39, P42\}$ at the base of both eyebrows and the top of both eyes, where it is normalized by dividing by the square of *L*.

**Area between eyebrows** The area between the eyebrows is that of the rectangle formed by connecting the four points $\{P21, P22, P39, P42\}$ at the base of the both eyebrows and the top of the both eyes, where it is normalized by dividing by the square of *L*.

**Area of eyes (right and left) ** This is the area of the hexagon formed by connecting six points around the perimeter of the right eye (left eye). To compute this area, we use the set $\{P36, P37, P38, P39, P40, P41 \}$ of points for the right eye ($\{P42, P43, P44, P45, P46, P47 \}$ for the left eye). The formula for calculating the area of the right eye consisting of these coordinates is defined by:

1 $$\begin{aligned} S=\frac{1}{2}\left| \sum _{j=36}^{41}\left( x_{j}-x_{j+1}\right) \times \left( y_{j}+y_{j+1}\right) \right| \end{aligned}$$

Finally, the area is normalised by dividing by the square of *L*.

**Eye aspect ratio (left to right)** To calculate the feature of eye aspect ratio, we refer to the points in Fig. 3. Let $L_v$ be the vertical length of the right (left) eye which is the length of the line segment connecting the top *P*11 (*P*39) and bottom *P*17 (*P*45) points on the right (left) eye, and $L_h$ the horizontal length of the right (left) eye which is the length of the line segment connecting the leftmost *P*8 (*P*36) and rightmost *P*14 (*P*42) points to the horizontal length of the right eye. The eye aspect ratio was then calculated using $L_v / L_h$.

**Blink rate** The number of times the eyes were closed during the 3 s immediately before classifying deception was measured. We considered that the eyes were closed if the area of the eyes was less than the first quartile in the entire data set of such areas.

**Eye movement (right and left)** To calculate the feature of eye movement, we refer to the points in Fig. 3. Horizontal eye movement is calculated as the distance from the inner corner *P*14 (*P*36) of the right (left) eye to the centre of iris of the eye, which is the average of points *P*23 and *P*27 (*P*51 and *P*55). Similarly, vertical eye movement is calculated as the distance from the top *P*11 (*P*39) of the right (left) eye to the centre of the iris of the eye which is the average of points *P*23 and *P*27 (*P*51 and *P*55).

**Mouth area (inside)** This is the area of the octagon formed by connecting the points $\{P60, P61, P62, P63,$ $P64, P65, P66, P67 \}$ on the inner perimeter of the mouth, where it is normalised by dividing by the square of *L*.

**Mouth area (outside)** This is the area of the dodecagon formed by connecting the points $\{P48, P49,$ $P50, P51, P52, P53, P54, P55, P56, P57, P58, P59\}$ on the outer perimeter of the mouth, which is normalized by dividing by the square of *L*.

**Mouth aspect ratio (inside)** Let $L_v$ be the vertical length of the mouth, which is the length of the line segment connecting the top *P*62 and bottom *P*66 points on the inner circumference of the mouth, and $L_h$ the horizontal length of the mouth, which is the length of the line segment connecting the leftmost *P*60 and rightmost *P*64 points of the mouse. Then, the inside mouth aspect ratio was calculated as $L_v / L_h$.

**Mouth aspect ratio (outside)** Similar to the inside mouth aspect ratio, the outside mouth ratio can be calculated using the points $\{P51, P57, P48, P54\}$.

**Degree of raise for mouth corners** This was calculated by subtracting the sum $y_v$ of the *y*-coordinates of the uppermost *P*51 and lowermost *P*57 points of the mouth from the sum $y_h$ of the *y*-coordinates of the rightmost *P*48 and the leftmost *P*54 points, where it was normalized by dividing by $y_v$. If the resultant value was positive, then the angle of the mouth increases.

**Angle of mouth** This was calculated by taking the average of the angles between the two line segments formed by connecting the set $\{P48, P49, P59\}$ ($\{P53, P54, P55\}$) of points around the rightmost (leftmost) on the periphery of the mouth.

**Number of times the mouth is closed** This is the number of times the mouth was closed within 3 s immediately before classifying deception. We treated the mouth as closed if the inner area of the mouth was less than the first quartile in the entire dataset of such areas.

**Gaze and head tilt** For both features, we used the values obtained from the OpenFace library as is.

**Pulse rate** We obtained the value of the pulse rate per second from a smartwatch. These values were written to a CSV file, and we adjusted the number of values to that of the frames in the recorded videos (30 frames per second) when we created the dataset.

### Preprocessing for dataset construction

#### Removing missing values

A certain number of values were missing in the pulse data acquired by the smartwatch. The presence of missing values made it difficult to accurately calculate the mean or standard deviation of the entire dataset. Therefore, missing values should be deleted or interpolated if they exist in the dataset. However, deleting all samples that contain missing values may result in wasted data or biased datasets. Even if we interpolate such samples, the resultant dataset may be biased, depending on the number of interpolated samples. In this study, because the confirmed number of missing values was rather small (about one sample per thousand), we excluded samples that contained missing values from our dataset.

#### Outlier removal

An outlier is the data that statistically far from the others in the same dataset.

An outlier is data that is statistically far from the others in the same dataset. If we leave the outliers as they are, they may distort the statistical indices during data analysis. Hence, we need to address outliers using measures or detection methods, depending on the type or cause of outliers. In this study, we regarded the data as an outlier and deleted it from our dataset if it was lower or higher than the first quartile and the third quartile, respectively, on the entire dataset from a statistical perspective.

#### Undersampling

Undersampling is a method of random data selection from the data of a majority group to match the number of data points in a minority group. When solving a classification problem using a machine learning model trained on unbalanced data, if the model is trained without any special treatment for balancing the data, the classification accuracy is lower for minority classes in the dataset.

If the purpose of the model is to classify the data of the majority group, this may not be a problem. However, in general, we also need to maintain high accuracy in classifying the data of minority groups. Thus, this issue must be addressed.

In this study, although the data for negative cases were used as they were, the data for positive cases were trimmed down to the range of 3 s before and 5 s after the deceptive utterance. Thus, negative cases comprised a minority of the total data samples.

Therefore, we reduced the amount of data on negative cases to match that of positive cases using undersampling.

### Classification by machine learning

Based on the preprocessed dataset that consisted of the abovementioned features and corresponding labels, we trained a machine learning model and evaluated the performance of the obtained models. In this study, we detect deception based on the ranking of feature importance.

As usual, we used accuracy, precision, recall, and F1 score as the performance metrics. To calculate these metrics, the confusion matrix presented in Table 2 was used.

**Table 2:** Confusion matrix

		Predictive class	
		Positive	Negative
Actual class	Positive	TP	FN
	Negative	FP	TN

accuracy is the fraction of correct predictions in all prediction results, defined as given below.

2 $$\begin{aligned} \text {Accuracy} = \frac{T P + T N}{T P + F P + F N + T N} \end{aligned}$$

Precision is the fraction of the number of data classified as positive in the number of data points that are actually positive, defined as

3 $$\begin{aligned} \text {Precision}=\frac{T P}{T P+F P} \end{aligned}$$

Recall is the metric of how well our model can classify relevant data.

4 $$\begin{aligned} \text{ Recall } =\frac{T P}{T P+F N} \end{aligned}$$

Finally, the F1 score (F value) is the metric by taking the harmonic mean of precision and recall.

5 $$\begin{aligned} \text {F1 score}=\frac{2 \text{ Recall } \cdot \text{ Precision } }{ \text{ Recall } + \text{ Precision } } \end{aligned}$$

To evaluate the generalization performance of the trained models, we conducted a 10-fold cross-validation. That is, we divided our dataset into 10 segments, then some samples as the testing dataset, and the others as the training dataset, and evaluated the performance of a trained model using the testing dataset. We repeated this process 10 times and changed the segment of the testing dataset for each trial. Finally, the generalization performance was calculated based on the average performance of these trials.

## Experiments

### Overview of our experiment

The purpose of this study was to develop a model for detecting deceptive statements made by interviewees in remote interviews and to investigate what features are helpful in classification. As mentioned in Sect. 3.1, we collected our raw dataset by taking an alternative approach that provides a similar (psychological) situation for our subjects to the actual interviews. Then, we evaluated the performance of machine learning models (RF) based on the above data using 10-fold cross-validation, and then investigated whether the model (or more important features) helped classify deception in actual (role-played) interviews.

In the latter experiment, the collected data from each subject was regarded as testing data, the performance was evaluated using the trained model, and the results were compared with those of the 10-fold cross-validation.

To compare the performance among the models, we mainly focused on the F1 score because we thought both precision and recall are important measures from the perspective of an interviewer who might have an interest in using an automatic deception detection system in real interviews.

To collect data from our subjects, we used a smart- watch Polar M600 to collect the pulse rate and a web camera Logitech C270n video data, where the frequency of recording video was 30 frames per second and the resolution of the video was $1280\times 720$. We implemented our experimental programs using the Python 3 programming language (specifically, the Anaconda 3 distribution, including the scikit-learn and matplotlib libraries for machine learning and visualization, respectively) and the OpenFace 2.0 library [1].

### Results on performance evaluation

In the experiment, we collected data from four male subjects, aged 23–25 years, who were graduate students at our institute (Tokyo University of Science). As mentioned in Sect. 3.4.3, we adjusted the amount of data on positive cases to that on negative cases using undersampling because the positives were trimmed down to a certain range on the time sequence, resulting in an imbalanced dataset (see Table 3).

**Table 3:** Number of data for each subject before and after undersampling

	# of original data		# of undersampled data	
	Positive	Negative	Positive	Negative
subject 1	4301	25,362	4301	4301
subject 2	2563	14,347	2563	2563
subject 3	9858	61,097	9858	9858
subject 4	2025	14,361	2025	2025

**Table 4:** Performance of our models using Random Forest

	Results evaluated by the 10-fold cross validation					Results tested by dataset of role-played interviewing			
	subject 1	subject 2	subject 3	subject 4		subject 1	subject 2	subject 3	subject 4
TP	3584	1914	6499	1850		873	453	659	516
TN	717	649	2459	175		187	194	236	94
FP	1213	499	1652	342		282	85	183	103
TN	3088	2064	7309	1683		809	550	714	502
Accuracy	0.78	0.78	0.77	**0**.**87**		0.78	0.78	0.77	**0**.**84**
Precision	0.75	0.8	0.8	**0**.**84**		0.76	**0**.**84**	0.78	0.83
Recall	0.83	0.75	0.73	**0**.**91**		0.84	0.7	0.74	**0**.**85**
F1 score	0.79	0.77	0.76	**0**.**88**		0.79	0.76	0.76	**0**.**84**

Bold text means the best result for each performance measure

Let us now consider the results of our experiment. On the left-hand side of Table 4, we show the performance of our models (RFs) after 10-fold cross-validation, where these models were trained using the data collected by the proposed method in Sect. 3.1.

Regarding accuracy, which simply measures whether deceptive statements can be detected, the result for Subject 4 showed the highest score of 0.86, whereas the others vary from 0.77 to 0.78. The results indicate that there was a difference in the ease with which the facial features appeared among the subjects and that detecting the facial expressions of Subject 4 was easier than the others. We also observed a similar trend in the F1 values.

As for precision and recall, recall tended to be higher than precision for Subjects 1 and 4, which implies that the coverage of detecting actual deceptive utterances made by them was slightly higher, but some overlooking of deception occurred. In contrast, the results for Subjects 2 and 3 had a higher precision than recall. That is, the model tended not to overlook deceptive utterances, but to have more false positives. For example, the model misclassified that the subject said a deception when he did not intend to deceive the interviewer but spoke while recalling an ambiguous memory about a provided image. In addition, the model misclassified when the subject spoke the truth immediately after the deceptive speech, which was due to the continuous stress on the subject.

Next, let us examine the results shown on the right side of Table 4. To obtain these results, we used exactly the same models evaluated by 10-fold cross-validation, but tested them using the dataset of role-played remote job interviews.^1^ There were no significant differences between these results and those on the left side of Table 4. Hence, we may claim that the proposed method for data collection forced subjects into almost the same (psychological) situation of job interviewing. It addition, it might be helpful in detecting deceptive statements of interviewers in similar applications in the real world.

### Investigation of feature importance

**Fig. 4:**
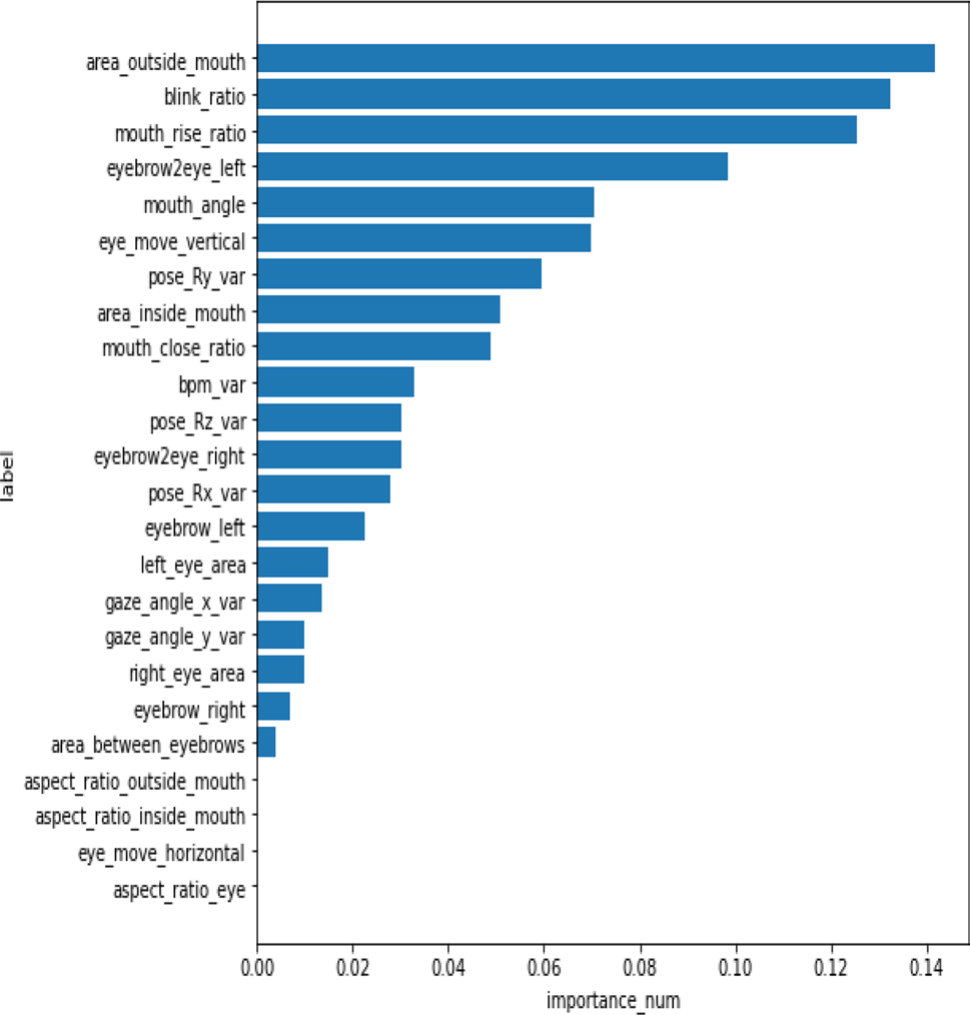
Importance of features for subject1

**Fig. 5:**
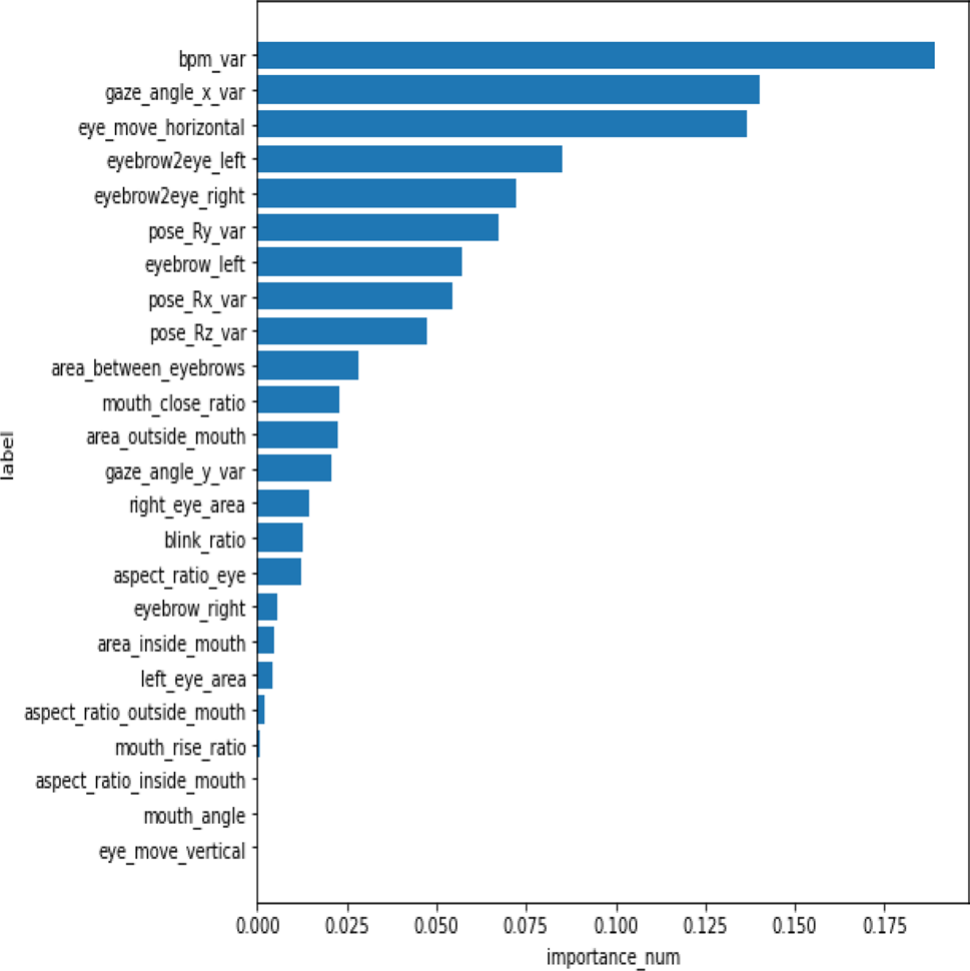
Importance of features for subject2

**Fig. 6:**
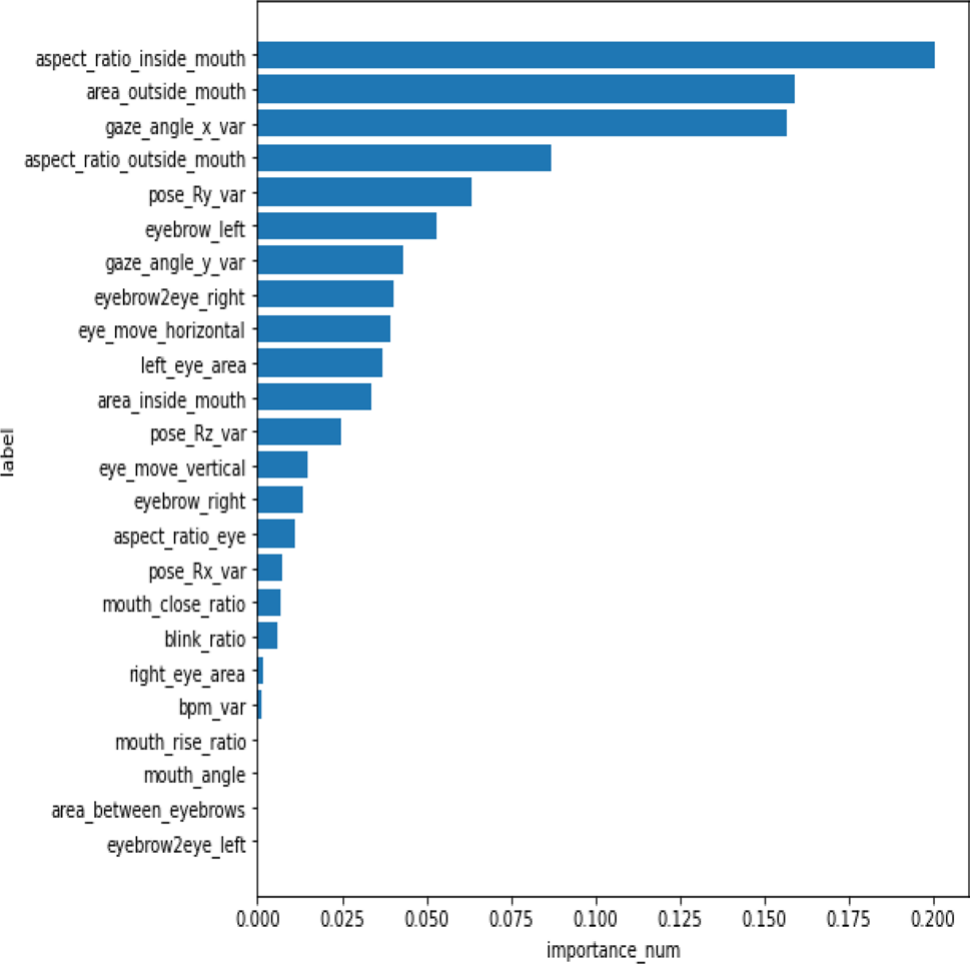
Importance of features for subject3

**Fig. 7:**
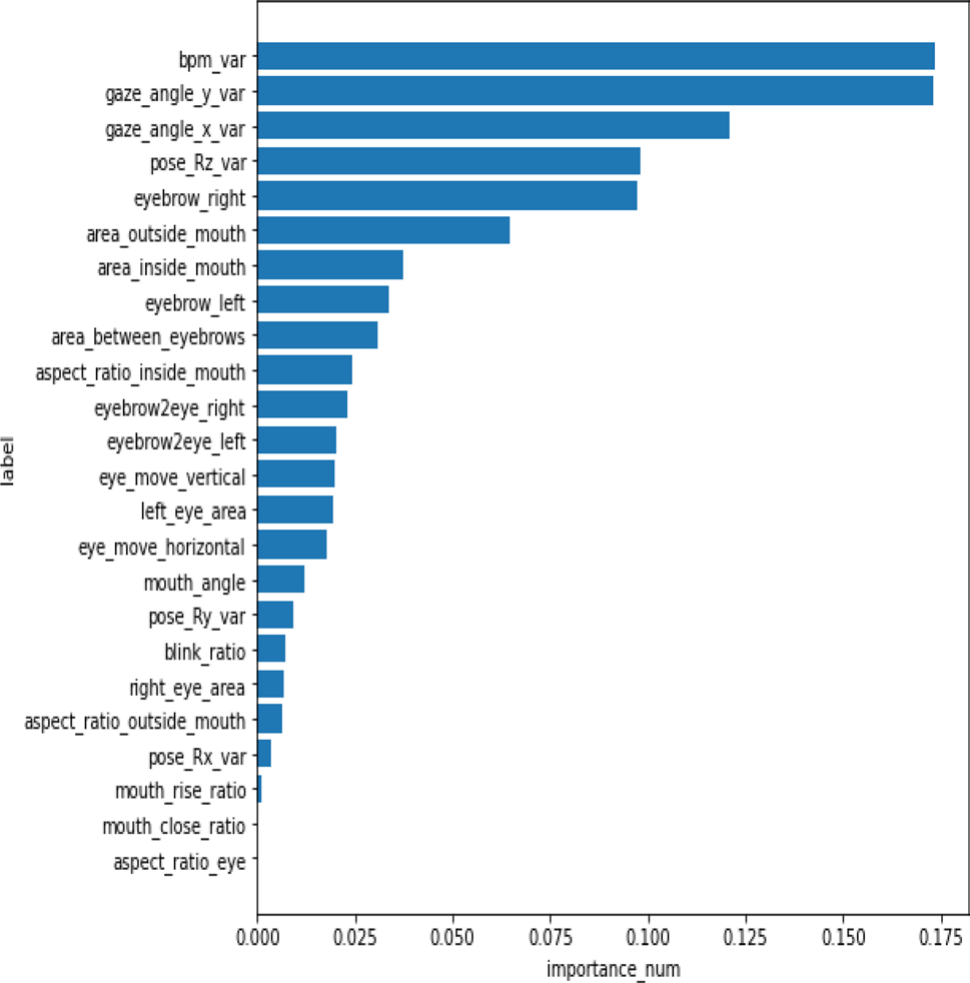
Importance of features for subject4

Figures 4, 5, 6 and 7 show the list of important features for each subject, where we visualized the trained models based on the dataset of our proposed method. That is, we did not use the testing dataset of role-played interviews to visualize the ranking of features. First, for Subject 1 in Fig. 4, the features of the inside and outside of the mouth areas and the number of blinks were particularly important to detect deceptive speech. A change in the area of the mouth might indicate that the subject closed their mouth when silent or opened it rather widely or narrowly when talking. Note that we could not observe that Subject 1 did not open his mouth widely according to the recorded video. In addition, the area between the eyebrows, vertical movement of the eyes, angle and aspect ratio of the mouth, and yaw rotation of the head also contributed to the classification to some extent. These features also reflected the deceptive behaviors of Subject 1 during our experiment; that is, he looked up (or down) while thinking about what he wanted to say during the deceptive speech, smiled involuntarily, and closed his mouth a little. The pulse rate could also contribute to the classification, although it is relatively smaller than that mentioned above. Hence, the deceptive behaviors of Subject 1 can be characterized by the actions of thinking, silence, and blinking, which involve pulse changes.

Similar to Subject 1 (Fig. 4), we also observed the characteristics of Subjects 2, 3, and 4, as follows. 

Subject 2: The top three important features in Fig. 5 are the variances of the pulse rate and the horizontal movement of the gaze and the head’s yaw rotation. The importance of pulse rate was particularly high, and we observed that his pulse rate changed significantly during his deceptive speech. Other important features include the distance between the left eyebrow and eye, the tilt of the right eyebrow, and the angle of the mouth. These results indicate that his pulse rate tended to increase during deceptive speech, and the changes in gaze and areas around his eyes, except for vertical movement, were relatively significant. In addition, the features of the mouth area were slightly less significant, which might imply that he could continuously speak deceptive speech, like truthful speech, but slightly changed the talking speed without much silence.

Subject 3: Fig. 6 shows that the inside/outside mouth aspect ratio, outside mouth area, and horizontal movement of the gaze were particularly important features. Then, the head yaw rotation and left eyebrow tilt were followed. However, in contrast to the above two subjects, features such as the variance of the pulse rate, the number of blinks, the number of mouths closed, and the angle of the mouth contributed less to detecting deception. Based on this observation, we expect that he tends to be whispered or silent if he becomes less confident during his deception.

Subject 4: Significant features in Fig. 7 are the pulse rate, the horizontal/vertical movement of the gaze, head roll rotation, tilt of the right eyebrow, and inside and outside of the mouth area. This indicates that the pulse rate, the direction of gaze, and head posture changed more during his reception.

Some of the expected characteristics among our subjects were observable according to our recorded videos, but many of them appeared or disappeared quickly. Therefore, our approach might be helpful for interviewers to support their intuition of detecting deception during interviewing.

## Conclusion

In this study, we have proposed a method to aid in the detection of deception based on machine learning, which excludes human bias from detection. We also presented a method to construct a more realistic dataset for the task of deception detection by asking subjects not to respond artificially but rather to improvise natural responses.

Our results on the experiment of 10-fold cross-validation using random forest classifiers based on extracted features from facial expressions and pulse rates showed that the accuracy and F1 value was in the range between 0.75 and 0.8 for each subject, and the highest ones were 0.87 and 0.88, respectively. Through the analysis of the feature importance of the trained models, we found that the characteristics of facial expressions during deception differed among subjects, which was reflected in their rankings. Fortunately, we observed some common features among two or three subjects, such as the area inside and outside of their mouth, the area around the eyes, movement of the gaze, and changes in pulse rate, although there was no such common feature that had extremely high importance (ranked in the top five) for all subjects.

In addition, the performance evaluation results that used the testing dataset of role-played job interviews showed almost similar performance to the result of the 10-fold cross-validation based on the dataset of the proposed method. Therefore, our method may help construct a dataset for detecting deception in the real world.

Further, we investigated an approach that uses binary classification to detect deception. However, we observed some cases in which the trained model predicted deception when the subject did not intend to deceive the interviewer but spoke while recalling an ambiguous memory about a provided image. Hence, there is room to extend our approach to handle multi-class classification that can correctly classify the cases where “the subject tells a truth,” “the subject tells a lie where they intended to deceive the interviewer,” and “the subject makes a false statement where they did not have the intention to deceive the interviewer, potentially owing to vague memories.”

Additionally, we did not consider the subjects’ personalities, whereas real organizations and companies often consider the results of personality tests, which is an earlier part of the interviewing process. Therefore, we may also extend our approach to include psychological assessments such as the Ten Item Personality Inventory [3] to analyze the relationship between the subjects’ psychological profiles and the results of the interviewing experiment, as a further study.

Finally, we must note the limitation of this study. In order to obtain statistically rigorous results using our approach, we essentially need several thousands of recorded videos and richer sensor data with the help of a huge number of subjects, who might have different cultural backgrounds and neurodivergent statuses, but it was difficult for us. Hence, we restricted our attention to providing some case-study style analysis with the help of few subjects based on the range of our proposed method in the present literature.

Our approach and results might be helpful for interviewers who might have an interest in using an automatic deception detection system in real interviews, where, as we noted in our introduction, they need to be careful with ethical issues and local laws when using such an application.

## Data Availability

Since preparing to open our dataset for the public takes much time, we would like to skip writing such a statement now.

## Footnotes

1 The reason why we selected remote job interviews here is that our subjects comprise students and can be relatively easier to simulate job hunters than the other roles such as a terrorist who wants to deceive border guards as we mentioned in the relevant studies.
